# Using Circulating Tumor DNA as a Novel Biomarker to Screen and Diagnose Colorectal Cancer: A Meta-Analysis

**DOI:** 10.3390/jcm12020408

**Published:** 2023-01-04

**Authors:** Liang Min, Jinghua Chen, Meihong Yu, Deliang Liu

**Affiliations:** 1 Department of Gastroenterology, The Second Xiangya Hospital of Central South University, Changsha 410011, China; 2 Research Center of Digestive Disease, Central South University, Changsha 410011, China; 3 Department of Oncology, The Third Xiangya Hospital of Central South University, Changsha 410013, China

**Keywords:** circulation tumor DNA, colorectal cancer, diagnosis, meta-analysis

## Abstract

(1) Background: Circulating tumor DNA (ctDNA) has emerged as a promising biomarker for many kinds of tumors. However, whether ctDNA could be an accurate diagnostic biomarker in colorectal cancer (CRC) remains to be clarified. The aim of this study was to evaluate the diagnostic accuracy of ctDNA in CRC. (2) Methods: PubMed, Web of Science, and Cochrane databases were searched to identify studies reporting the use of ctDNA to screen and diagnose CRC, and all relevant studies published until October 2022 were enrolled for our analysis. These studies were divided into three primer subgroups: the subgroup of quantitative or qualitative analysis of ctDNA and the subgroup of septin9 (SEPT9) methylation assay. (3) Results: A total of 79 qualified articles with 25,240 subjects were incorporated into our meta-analysis. For quantitative studies, the combined sensitivity (SEN), specificity (SPE), and diagnostic odds ratio (DOR) were 0.723 (95% CI: 0.623–0.803), 0.920 (95% CI: 0.827–0.966), and 23.305 (95% CI: 9.378–57.906), respectively, yielding an AUC of 0.860. The corresponding values for qualitative studies were 0.610 (95% CI: 0.566–0.651), 0.891 (95% CI: 0.878–0.909), 12.569 (95% CI: 9.969–15.848), and 0.823, respectively. Detection of SEPT9 methylation depicted an AUC of 0.879, with an SEN of 0.679 (95% CI: 0.622–0.732), an SPE of 0.903 (95% CI: 0.878–0.923), and a DOR of 20.121 (95% CI:14.404–28.106), respectively. (4) Conclusion: Blood-based ctDNA assay would be a potential novel biomarker for CRC screening and diagnosis. Specifically, quantitative analysis of ctDNA or qualitative analysis of SEPT9 methylation exhibited satisfying diagnostic efficiency. Larger sample studies are needed to further confirm our conclusions and to make the ctDNA approach more sensitive and specific.

## 1. Introduction

Colorectal cancer (CRC) is the third most commonly diagnosed cancer and the second most common cause of cancer death worldwide, which accounts for nearly 10% of cancer cases and 9% of cancer deaths [1]. Even though there have been dramatic advances in understanding the molecular mechanism and clinical treatment over decades, helping to double the overall survival of advanced CRC to three years, there was estimated over 0.9 million CRC-related death in 2020 [2]. Since there are significant differences in prognosis and therapy response between different clinical stages in CRC patients, it is extremely important to diagnose and treat CRC at an early stage [3]. However, most CRC patients are asymptomatic at an early stage. When common symptoms such as constipation, bleeding, anemia, or abdominal pain are noted, CRC may already develop to advanced stages [4]. Currently, several methods, including fecal occult blood test (FOBT), fecal immunochemical test (FIT), and colonoscopy, have been applied in CRC screening, which significantly reduced CRC mortality [5]. However, there are some defects in the current screening method. The sensitivity (SEN) and specificity (SPE) of FOBT and FIT for early CRC are unsatisfactory [6,7]. Additionally, the FIT demonstrated poorer compliance in CRC monitoring compared to other simple methods, such as blood-based tests [8,9]. Although colonoscopy has been regarded as the gold standard for CRC screening, it has low acceptance by general people due to the invasive procedure, troublesome diet restriction, and bowel preparation [10,11]. Moreover, a colonoscopy might not reach the entire population in developing countries as it requires special instruments and qualified endoscopists to which they have limited access. Therefore, a simple, noninvasive, and cost-efficienct approach is urgently needed to screen and diagnose CRC.

Since the detection of higher levels of circulating free DNA (cfDNA), which consists of fragments of extracellular, in the blood of patients with various types of cancer [12], accumulating evidence has demonstrated that cfDNA could serve as a promising biomarker in multiple kinds of disease [13]. While most cfDNA originates from the hematopoietic system in a healthy person [14], apoptotic or necrotic cancer cells release DNA called circulating tumor DNA (ctDNA) into the biological fluid in cancer as a small proportion of cfDNA in cancer patients [15,16]. Although ctDNA may only account for less than 1% of cfDNA [17], ctDNA has been extensively explored with improving assay techniques. The analysis of ctDNA has been regarded as a promising tool in oncology as it can be detected by noninvasive methods and provide cancer-specific information, including nucleotide mutation [18], copy number aberration (CNA) [19], DNA methylation [20], and microsatellite instability (MSI) [21].

Alteration of ctDNA has been detected in CRC patients by the quantitative method, which measures the quantity of ctDNA [22], and the qualitative method, by reporting cancer-specific changes, including DNA methylation, can also detect alteration of ctDNA in CRC patients [23]. Although accumulating evidence indicated that ctDNA is a promising biomarker in CRC screening and diagnosis, there are some concerns about the application of ctDNA in CRC diagnosis due to the tumor heterogeneous, unstandardized protocol and concentration threshold [24]. Therefore, it is necessary to conduct a comprehensive analysis and evaluation of the diagnostic values of ctDNA in CRC before large-scale clinical application. Thus, we implemented a meta-analysis to assess the diagnostic performance of ctDNA assays for CRC, which could potentially contribute to the technology development and modify the clinical decision.

## 2. Materials and Methods

### 2.1. Protocol and Registration

This systematic review and meta-analysis were performed according to the Preferred Items for Systematic Review and Meta-Analysis (PRISMA) [25] and Meta-analysis Of Observational Studies in Epidemiology (MOOSE) group [26]. The study was also registered at the International Prospective Register of Systematic Reviews (PROSPERO) database (Number: CRD42022370118).

### 2.2. Search Strategy

PubMed, Web of Science and The Cochrane Library were searched with query term: “(circulating tumor DNA OR circulating DNA OR ctDNA OR Cell Free Tumor DNA OR Plasma DNA OR Serum DNA) AND (Colonic Neoplasm OR Colon Neoplasm OR Colorectal cancer OR Colorectal Carcinoma OR Colonic Carcinoma OR Colonic Cancer OR Colon Cancer OR Colonic tumor OR Colon tumor) AND (Diagnosis OR Detection OR Screen OR Sensitivity OR Specificity)”. The language was limited to English. All potentially relevant articles published until October 2022 were retrieved and independently queried by two authors. We also manually screened the reference from the included studies and relevant reviews for enlarged retrieval.

### 2.3. Inclusion and Exclusion Criteria

The articles that conformed to the following criteria were included: (a) ctDNA was used for first-diagnosed CRC patients rather than the recurrent patients; (b) the numerical value of SEN and SPE of each detection method could be calculated in each study; (c) specimens were extracted from peripheral blood. The exclusion criteria were as follows: (a) review, case report or case series, comments, letter, or conference abstract; (b) sample size of studies was less than 10; (c) duplicated or overlapping publications that utilized the same patient specimens and identical gene modification; (d) publications with no full-text available. Two authors independently evaluated the eligibility of all publications. A third author would assess studies when there was a discrepancy.

### 2.4. Data Extraction

Data from eligible publications were extracted and compiled by two independent authors. The information extracted from included studies was as follows: the first author’s name, publication year, region/country, study design, sample size, control type, source of specimens, sampling time, detection method, reference gene, cut-off values, diagnostic performance (including SEN and SPE), true positive (TP), true negative (TN), false positive (FP), false negative (FN), positive likelihood ratio (PLR) and negative likelihood ratio (NLR), and diagnostic concordance.

### 2.5. Quality Assessment

The quality of all included studies was evaluated by two independent authors according to Quality Assessment of Diagnostic Accuracy Studies-2 (QUADAS-2), which assesses the studies on four key domains, including patient selection, index test, reference standard, and flow timing [27]. Two authors independently labeled “low risk”, “some concerns”, and “high risk” for each domain in each study, and divergence was discussed until an agreement was reached. Publications would be excluded if they were regarded as poor quality by both authors.

### 2.6. Statistical Analysis

We utilized Revman Manager 5.4 and R program (R Foundation, Vienna, Austria, version 4.2.1) with “meta” [28], “mada” [29], and “metafor” [30] packages to conduct this diagnostic meta-analysis. The pooled sensitivity (SEN), specificity (SPE), positive likelihood ratio (PLR), positive likelihood ratio (NLR), diagnostic odds ratio (DOR), and corresponding 95% confidence interval (95% CI) were calculated as an indicator of test performance [31]. The summary receiver operating characteristic (SROC) curve and corresponding area under the curve (AUC) were formulated to assess the overall test accuracy [32,33]. Generally, the AUC ranges of 0.7–0.8, 0.8–0.9, and 0.9–1.0 were interpreted as acceptable, excellent, and outstanding accuracy, respectively [34,35]. The Spearman correlation coefficient and corresponding *p*-value were used to identify the presence of the threshold effect [36]. Heterogeneity among the studies was evaluated by chi-square and I^2^ tests. *p* < 0.10 or I^2^ > 50% indicated a significant heterogeneity, and the random effect model should be used [37]. Subgroup analysis and meta-regression were conducted to further reveal the source of heterogeneity [38]. Potential publication bias was checked by Deek’s funnel plot asymmetry test [39].

## 3. Results

### 3.1. Studies Characteristics

**Figure 1:**
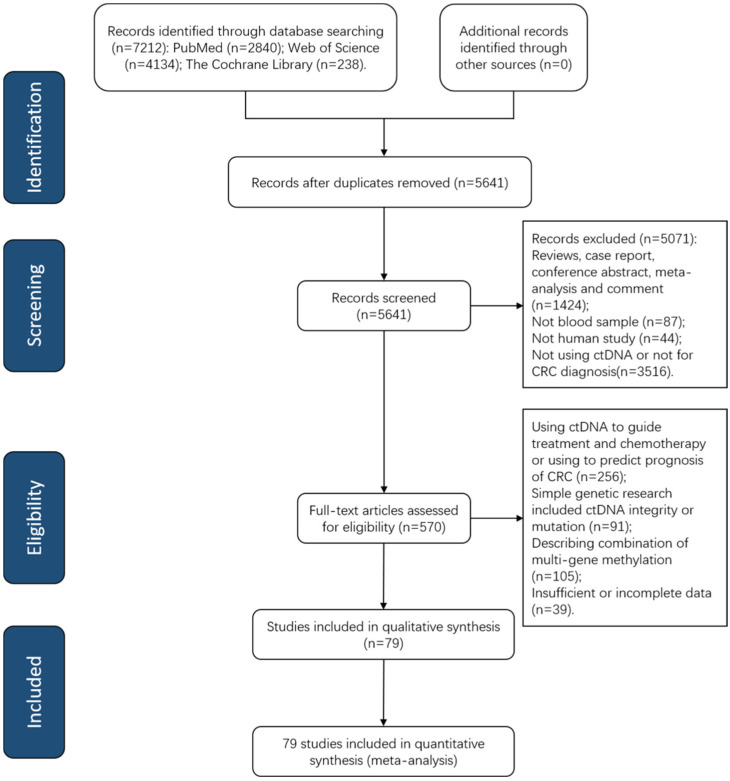
A PRISMA flow diagram of paper search. CRC, colorectal cancer; ctDNA, circulating tumor DNA; PRISMA, Preferred Reporting Items for Systematic Reviews and Meta-Analysis.

Figure 1 shows a PRISMA flow diagram depicting the retrieval strategy of databases to incorporate qualified studies. A total of 7212 publications were queried based on our search strategy at first, and 79 eligible articles published from 2008 to 2022 were incorporated into our meta-analysis after examination of the abstract and comprehension of the full text (Table S1). All included studies consisted of quantitative analysis of ctDNA concentration (*n* = 11) [40,41,42,43,44,45,46,47,48,49,50] and qualitative analysis of tumor-specific gene methylation in ctDNA (*n* = 67) [51,52,53,54,55,56,57,58,59,60,61,62,63,64,65,66,67,68,69,70,71,72,73,74,75,76,77,78,79,80,81,82,83,84,85,86,87,88,89,90,91,92,93,94,95,96,97,98,99,100,101,102,103,104,105,106,107,108,109,110,111,112,113,114,115,116,117,118] as well as both quantitative and qualitative analysis (*n* = 1) [119]. Among these publications, 34 articles [51,59,60,62,63,64,65,66,67,68,70,71,72,74,75,78,79,81,82,83,90,91,96,100,101,102,104,105,107,108,110,112,115,116] evaluated the diagnostic efficiency of septin9 (SEPT9) methylation for CRC.

In our meta-analysis, a total population of 8076 CRC patients and 17164 control subjects were included. The primary characteristics of all studies are summarized in Table 1. The majority of participants were from the Asia area (*n*= 13,841). There were 18 prospective studies and 8 retrospective studies, while 53 studies did not clarify the study design. Among 50 studies with known sample collecting time, all samples were collected before surgery or treatment. While the majority of studies collected plasma for ctDNA analysis (*n* = 55), serum specimens were also analyzed in some research (*n* = 24). The methods applied to measure the concentration of ctDNA include real-time quantitative polymerase chain reaction (RT-qPCR) (*n* = 8), droplet digital polymerase chain reaction (ddPCR) (*n* = 2), branched DNA assay (bDNA) (*n* = 1) and florescent dye assay (*n* = 1). For qualitative analysis of ctDNA, methylation-specific polymerase chain reaction (MSP) was the most common method applied (*n* = 64).

### 3.2. Quality Assessment

**Figure 2:**
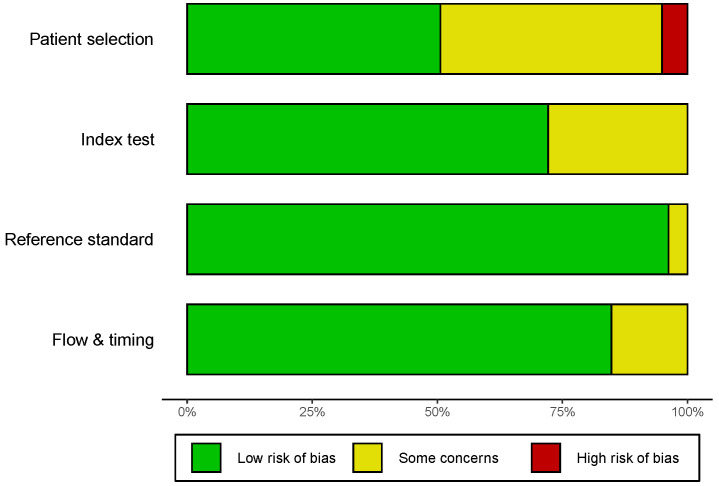
Quality assessment of the included studies by the revised QUADAS-2. QUADAS-2, Quality Assessment of Diagnostic Accuracy Studies-2.

The quality assessment was based on QUADAS-2, and the outcome of eligible studies is depicted in Figure 2 and Figure S1. The majority of incorporated studies possessed a moderate–high quality, indicating that the overall quality of the included studies was highly acceptable. However, there were some concerns about the patient selection of 39 studies, such as whether the patient selection was consecutive or random in a specific time period. Moreover, predefined threshold information was absent in 22 studies, which potentially generated some concerns about the index test. All enrolled patients obtained definite pathological diagnoses.

### 3.3. Diagnostic Value of Quantitative and Qualitative Analysis of ctDNA for CRC

**Figure 3:**
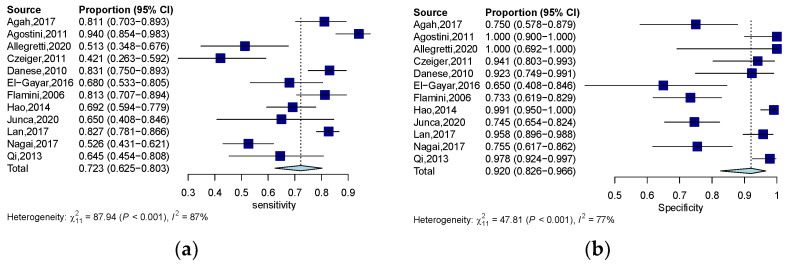
Forest plots of SEN (**a**) and SPE (**b**) for the diagnostic value of ctDNA assay for CRC in the quantitative detection subgroup. 95% CI, 95% confidence interval; CRC, colorectal cancer; ctDNA, circulating tumor DNA; SEN, sensitivity; SPE, specificity; diamond shapes representing average effect; dark blue squares representing individual study results.

**Figure 4:**
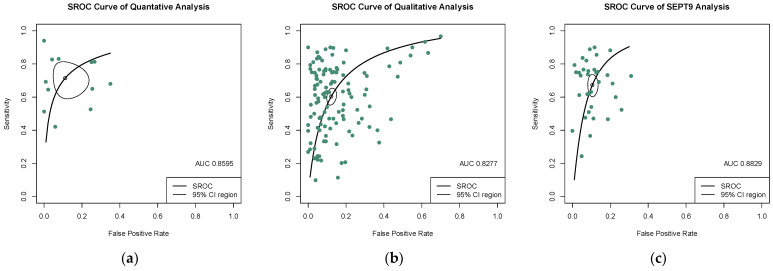
SROC curves of diagnostic value for (**a**) the quantitative detection subgroup; (**b**) the qualitative detection subgroup; (**c**) the SEPT-9 methylation detection subgroup. 95% CI, 95% confidence interval; AUC, the area under the curve; SROC, summary receiver operating characteristic.

The quantitative analysis group, including 12 studies, yields an SEN of 0.723 (95% CI: 0.623–0.803) and an SPE of 0.920 (95% CI: 0.827–0.966) (Figure 3a,b). The numeric value of PLR, NLR, and DOR were 6.820 (95% CI: 3.335–12.800), 0.326 (95% CI: 0.227–0.446), and 23.305 (95% CI: 9.380–57.906) respectively. The corresponding SROC yield an AUC of 0.860, indicating excellent accuracy in discriminating CRC patients from control subjects (Figure 4a). Notably, the significant heterogeneity among these quantitative studies was observed (SEN: I^2^ = 87.5%, *p* = 0.000; SPE: I^2^ = 77.0%, *p* = 0.000; DOR: I^2^ = 82.3%, *p* = 0.000). The Spearman correlation coefficient was 0.008 (*p* = 0.983), indicating that heterogeneity among quantitative studies was derived from other factors rather than the threshold effect.

**Figure 5:**
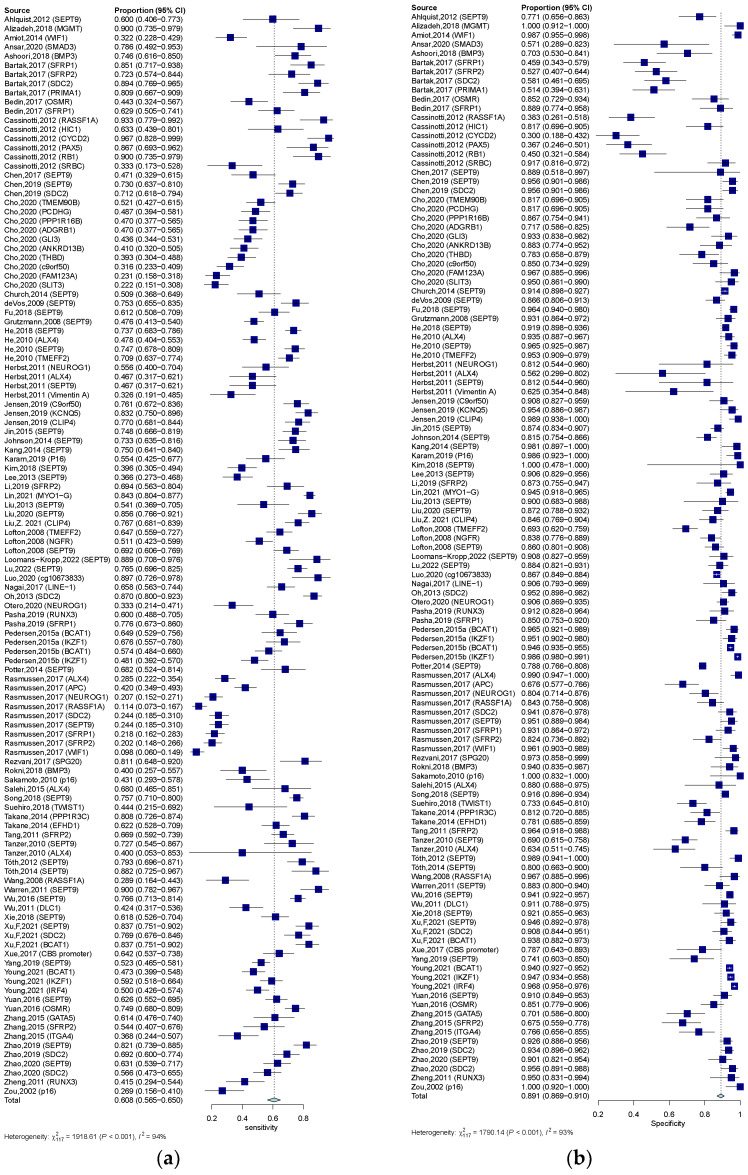
Forest plots of SEN (**a**) and SPE (**b**) for diagnostic value of ctDNA assay for CRC in the qualitative detection subgroup. 95% CI, 95% confidence interval; CRC, colorectal cancer; ctDNA, circulating tumor DNA; SEN, sensitivity; SPE, specificity; diamond shapes representing average effect; darkblue squares representing individual study results.

In the qualitative analysis group, an SEN of 0.609 (95% CI: 0.563–0.650), an SPE of 0.891 (95% CI: 0.864–0.910) (Figure 5a,b), and a DOR of 12.621 (95% CI: 10.004–15.922) were generated. The numeric values of PLR and NLR were 4.960 (95% CI: 4.190–5.860) and 0.453 (95% CI: 0.406–0.498). An AUC of 0.828 was calculated from the corresponding SROC, indicating excellent accuracy in discriminating CRC patients from control subjects (Figure 4b). Significant heterogeneity among these qualitative studies was also observed (SEN: I^2^ = 93.9%, *p* = 0.000; SPE: I^2^ = 93.5%, *p* = 0.000; DOR: I^2^ = 89.6%, *p* = 0.000). The Spearman correlation analysis was 0.149 (*p* = 0.118), depicting that heterogeneity among quantitative studies was derived from a non-threshold effect.

**Figure 6:**
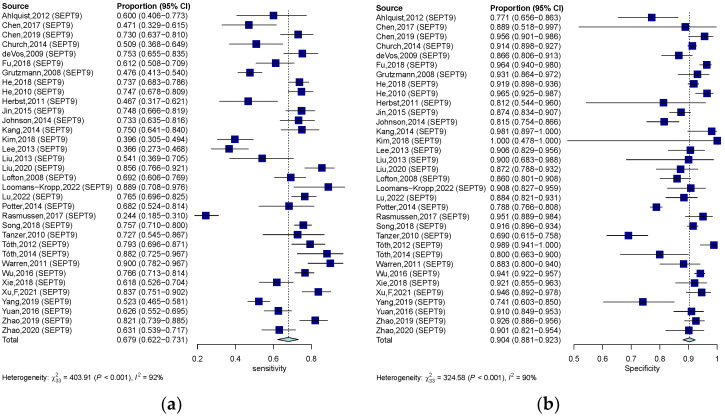
Forest plots of SEN (**a**) and SPE (**b**) for diagnostic value of ctDNA assay for CRC in the SEPT-9 methylation detection subgroup. 95% CI, 95% confidence interval; CRC, colorectal cancer; ctDNA, circulating tumor DNA; SEN, sensitivity; SPE, specificity; diamond shapes representing average effect; dark blue squares representing individual study results.

Methylation of SEPT9 was the most frequently detected epigenetic change in the qualitative analysis of ctDNA in CRC. Therefore, we also analyzed the diagnostic accuracy of SEPT9 methylation in discriminating CRC subjects from control. In 34 studies that measured the methylation of SEPT9, the pooled SEN, SPE, and DOR were 0.679 (95% CI: 0.622–0.732), 0.904 (95% CI: 0.881–0.923) (Figure 6a,b), and 20.551 (95% CI: 14.684–28.760), respectively. The pooled PLR and NLR were 6.420 (95% CI: 5.110–7.970) and 0.367 (95% CI: 0.307–0.430). The corresponding SROC yield an AUC of 0.883, indicating excellent accuracy in discriminating CRC patients from control subjects (Figure 4c). Significant heterogeneity among these studies was also observed (SEN: I^2^ = 91.8%, *p* = 0.000; SPE: I^2^ = 89.8%, *p* = 0.000; DOR: I^2^ = 82.3%, *p* = 0.000). The result of Spearman correlation analysis was 0.02 (*p* = 0.993), indicating that heterogeneity among quantitative studies was derived from the non-threshold effect (Table 1).

### 3.4. Subgroup Analysis and Meta-Regression Analysis

Different covariates including region (Asia vs. non-Asia), sample size (≥100 vs. <100), control type (health control vs. non-CRC disease), sample source (plasma vs. serum), assay methods (RT-PCR vs. other methods in quantitative analysis; MSP vs. other methods in qualitative analysis), and methylation gene (SEPT9 vs. other genes in qualitative studies) were applied in the subgroup analysis (Table 2).

Subgroup analysis based on sample size revealed an improved diagnostic accuracy in quantitative studies with sample size ≥ 100 since the SEP, SEN, and AUC for quantitative studies with sample size ≥100 were 0.771 (95% CI: 0.681–0.842), 0.924 (95% CI: 0.815–0.971) and 0.878, whereas corresponding values were 0.545 (95% CI: 0.417–0.667), 0.904 (95% CI: 0.604–0.983), and 0.694 for quantitative studies with sample size < 100. Similarly, qualitative analysis of ctDNA with a large sample size also had a more robust diagnostic accuracy (SEN: 0.598 (95% CI: 0.552–0.643), SPE: 0.903 (95% CI: 0.884–0.919), AUC: 0.849) compared to qualitative studies with sample size < 100 (SEN: 0.662 (95% CI: 0.544–0.762), SPE: 0.819 (95% CI: 0.694–0.900), AUC: 0.759).

For the quantitative analysis of ctDNA, subgroup analysis associated with control type showed that the SEN, SPE, and AUC for quantitative analysis of ctDNA to discriminate CRC from healthy control were 0.723 (95% CI: 0.626–0.803), 0.927 (95% CI: 0.833–0.967) and 0.861, while the corresponding values for quantitative analysis of ctDNA to discriminate CRC form non-CRC disease were 0.647 (95% CI: 0.508–0.765), 0.878 (95% CI: 0.563–0.976), and 0.641, respectively. While subgroup analysis in qualitative studies depicted an SEN of 0.601 (95% CI: 0.554–0.647), an SPE of 0.915 (95% CI: 0.895–0.932), and an AUC of 0.844 in qualitative studies with healthy people as control, and an SEN of 0.672 (95% CI: 0.617–0.723), an SPE of 0.809 (95% CI: 0.737–0.865) and an AUC of 0.774 in qualitative studies used non-CRC disease as control. These results indicated that studies utilizing healthy people as control provided a more satisfactory diagnostic efficiency compared to studies that used non-CRC patients as a control in both quantitative and qualitative analysis of ctDNA, which highlighted a more robust capability of ctDNA assay to distinguish CRC from healthy individuals than from non-CRC disease patients. Multiple levels of meta-regression also revealed that the parameter “control type” could contribute to the heterogeneity (Table 3). Moreover, heterogeneity was also derived from the parameter “methylation gene” as the corresponding coefficient was 0.661 (*p* = 0.01), which was consistent with the primary outcome of a clinical trial of SEPT9 methylation for CRC detection, indicating SEPT9 as a promising CRC diagnostic biomarker.

### 3.5. Publication Bias

**Figure 7:**
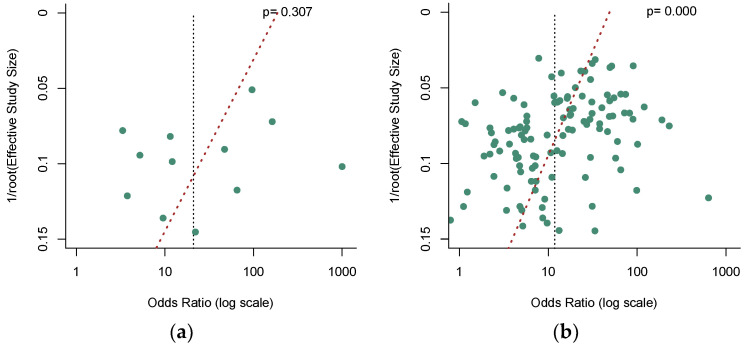

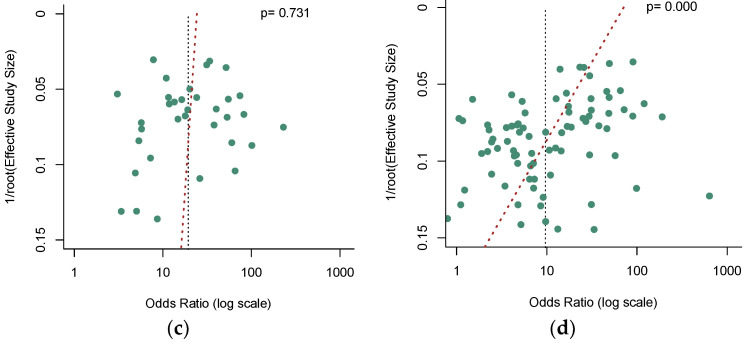
Funnel plots to evaluate the publication bias for (**a**) the quantitative detection subgroup; (**b**) the qualitative detection subgroup; (**c**) the SEPT-9 methylation detection subgroup; (**d**) the non-SEPT-9 methylation detection subgroup.

Deek’s funnel plot asymmetry test [39] was used to check the potential publication bias, and a *p*-value less than 0.05 indicated significance. No significant publication bias was observed in the quantitative analysis of ctDNA (Figure 7a, *p* = 0.307). Nevertheless, our results revealed a significant publication bias in the qualitative analysis of ctDNA (Figure 7b, *p* = 0.000). In addition, among these qualitative analysis studies, our analysis demonstrated that no significant publication bias existed in the SEPT9 methylation group (Figure 7c, *p* = 0.731), while a significant publication bias was found in the non-SEPT9 methylation group (Figure 7d, *p* = 0.000).

## 4. Discussion

CRC is one of the most common malignancies with poor prognosis and high mortality, which is highly correlated with the low early diagnostic rate [1]. When detected at an early stage, the survival rate of CRC would be over 90%, compared to approximately 7% for late-stage disease [120]. Although colonoscopy has demonstrated perfect diagnostic efficiency, it has low acceptance by general people due to the invasive procedure, troublesome diet restriction, and bowel preparation [10,11]. Thus, novel noninvasive diagnostic methods with robust diagnostic efficiency are in urgent need. Advance in precise ctDNA detection has made it possible for “liquid biopsy” of ctDNA to become a potential diagnostic biomarker of CRC [121]. In this diagnostic meta-analysis, we aimed to incorporate the diagnostic accuracy of ctDNA for CRC.

In our meta-analysis, quantitative analysis of ctDNA exhibited a higher SEN (0.723 vs. 0.609) and a comparable SPE (0.920 vs. 0.891) compared to qualitative analysis, which is consistent with a higher AUC (0.860 vs. 0.828). This might be explained by the fact that some genetic loci selected for the test were not commonly expressed in CRC, which might generate a false negative in the test. Various septins have been associated with tumorigenesis, and SEPT9 hypermethylation has been detected in CRC [122]. Furthermore, the detection of SEPT9 methylation in cfDNA has been regarded as a potential biomarker for CRC diagnosis and has become the first blood-based CRC screening test approved by the FDA [123]. When we looked into the SEPT9 methylation analysis among the qualitative analysis, the corresponding AUC was 0.883, relatively higher than the AUC of the quantitative analysis, with the value of the SEN (0.679) and SPE (0.904) similar compared to the quantitative analysis. These results depicted the superiority of SEPT9 methylation detection over other gene locus analyses in CRC diagnosis.

DOR was also applied to evaluate the diagnostic accuracy, and the discriminatory test performance would be classified as satisfactory when the value of DOR was over 10 [31]. Both quantitative and qualitative analyses of ctDNA exhibited satisfactory discriminatory test performance as the value of DOR was 23.305 and 12.621, respectively. Specifically, SEPT9 methylation assay among qualitative analysis revealed a higher DOR of 20.551 than that of qualitative analysis of ctDNA, indicating a better discriminatory test performance of SEPT9 methylation assay than other single gene methylation assay in qualitative analysis.

In this study, the value of PLR of quantitative analysis and SEPT9 methylation were 6.820 and 6.420, respectively, indicating that CRC subjects have an approximately six to seven folds higher chance of being positive in quantitative analysis and SEPT9 methylation analysis of ctDNA. However, qualitative analysis of ctDNA depicted a PLR of 4.920, which is lower than the value of quantitative analysis and SEPT9 methylation. Moreover, the qualitative analysis of ctDNA yielded an NLR of 0.496, implying that the probability for cases with negative qualitative assay results to have CRC is 49.6%, whereas the value of NLR of quantitative analysis and SEPT9 methylation detection of ctDNA were 0.326 and 0.367, respectively. These results might be explained that some gene locus methylation might not exist in all CRC cases, and using single gene methylation as a biomarker might lead to false negatives. However, SEPT9 methylation demonstrated a comparable PLR and NLR with quantitative analysis of ctDNA, depicting the robust diagnostic performance of SEPT9 methylation assay. Additionally, the SEPT9 assay demonstrated better compliance than FIT and colonoscopy in CRC screening [8,9].

Publication bias was evaluated by Deek’s funnel plot asymmetry test, and no publication bias was revealed in the quantitative analysis of ctDNA (*p* = 0.307). However, it was highly possible that publication bias was presented in the qualitative analysis group (*p* = 0.000). As previous analysis demonstrated the disparity between the SEPT9 methylation assay and other gene methylation assays within the qualitative analysis, we further evaluated the publication bias based on gene loci. The Deek’s funnel plot asymmetry test revealed that publication biases were likely to exist in the non-SEPT9 methylation assay (*p* = 0.000) but not in the SEPT9 methylation assay (*p* = 0.731). Furthermore, a meta-regression analysis was adapted to explore the potential source of heterogeneity. In the quantitative analysis of ctDNA, none of the parameters of region, control types, sample size, ample source, and assay methods represented a primary source of heterogeneity; thus, heterogeneity might be generated from subjects’ ages, TMN stages, tumor size, tumor metastasis, or different protocol, which failed to be evaluated in this meta-analysis due to the lack of complete information. In the qualitative analysis of ctDNA, among parameters of region, control types, sample size, ample source, assay methods, and gene loci methylation, “control type” (coefficient = 0.502, *p* = 0.034) and “gene loci methylation” (coefficient = 0.661, *p* = 0.010) were potential primary sources of heterogeneity. We further analyzed the heterogeneity in SEPT9 methylation studies and revealed that “region” (coefficient = 0.676, *p* = 0.045) and “control type” (coefficient = 0.733, *p* = 0.035) could be the primary sources of heterogeneity. Thus, further large clinical trials should reasonably select the control subjects and various ethnicities or be performed in multiple regions to boost the diagnostic performance of ctDNA of CRC.

Remarkably, there were several limitations that should be discussed in this meta-analysis. Firstly, even though we searched various databases, there were several publications we did not include in our meta-analysis as we failed to access the full texts. Moreover, there were only a small number of studies that described quantitative analysis of ctDNA, which might diminish the statistical significance. Thirdly, we failed to include some parameters, such as TMN stages, as incomplete information in included articles. Additionally, potential publication bias within the qualitative analysis that detects non-SEPT9 methylation, which might diminish the confidence of the analysis. Eventually, single gene loci were analyzed in qualitative analysis, while the combination of different gene alterations or other detection techniques might help to improve the diagnostic performance [124]. Therefore, more large-scale clinical trials with complete information should be performed to delineate the diagnostic value of ctDNA detection for CRC to further clarify the conclusions in this meta-analysis.

## 5. Conclusions

In summary, this is the first integrated meta-analysis on the overall diagnostic accuracy of circulating tumor DNA assays in CRC. Both quantitative and qualitative analyses of ctDNA exhibited excellent diagnostic efficiency. Specifically, quantitative analysis of ctDNA or qualitative analysis of SEPT9 methylation could potentially serve as effective diagnostic methods for CRC. Larger sample studies are needed to further confirm our conclusions and to make the ctDNA approach more sensitive and specific.

## Figures and Tables

**Table 1:** Summary of diagnostic accuracy of ctDNA assay for CRC in multiple subgroups.

Group	SEN (95% CI)	SPE (95% CI)	PLR (95% CI)	NLR (95% CI)	DOR (95% CI)	AUC
Quantitative analysis of ctDNA	0.723 (0.623–0.803)	0.920 (0.827–0.966)	6.820 (3.335–12.800)	0.326 (0.227–0.446)	23.305 (9.378–57.906)	0.860
Qualitative analysis of ctDNA	0.609 (0.563–0.650)	0.891 (0.864–0.910)	4.960 (4.190–5.860)	0.453 (0.406–0.498)	12.621 (10.004–15.922)	0.828
SEPT9 methylation assay	0.679 (0.622–0.732)	0.904 (0.881–0.923)	6.420 (5.110–7.970)	0.367 (0.307–0.430)	20.551 (14.684–28.760)	0.883

Abbreviations: 95% CI, 95% confidence interval; AUC, the area under the curve; CRC, colorectal cancer; ctDNA, circulating tumor DNA; DOR, diagnostic odds ratio; NLR, negative likelihood ratio; PLR, positive likelihood ratio; SEN, sensitivity; SPE, specificity.

**Table 2:** Subgroup analysis of diagnostic performance of ctDNA assay for CRC.

Analysis	Group	Subgroup	SEN (95% CI)	SPE (95% CI)	DOR (95% CI)	AUC
Quantitative analysis	Control type	HC	0.723 (0.626–0.803)	0.927 (0.833–0.967)	24.751 (9.704–63.129)	0.861
		NCD	0.647 (0.508–0.765)	0.878 (0.563–0.976)	13.444 (1.37–131.721)	0.641
	Sample size	≥100	0.771 (0.681–0.842)	0.924 (0.815–0.971)	32.219 (10.540–98.491)	0.878
		<100	0.545 (0.417–0.667)	0.904 (0.604–0.983)	6.753 (2.534–17.998)	0.694
	Sample source	Plasma	0.747 (0.584–0.861)	0.908 (0.735–0.972)	22.297 (4.819–103.165)	0.868
		Serum	0.701 (0.587–0.795)	0.929 (0.793–0.978)	25.591 (7.815–83.805)	0.841
	Assay method	RT-PCR	0.782 (0.680–0.853)	0.907 (0.767–0.967)	28.510 (7.995–101.662)	0.874
		Other	0.542 (0.445–0.635)	0.942 (0.786–0.986)	16.431 (4.418–61.105)	0.663
Qualitative analysis	Region	Asia	0.623 (0.574–0.669)	0.906 (0.886–0.922)	15.968 (11.843–21.529)	0.862
		Other	0.594 (0.519–0.665)	0.869 (0.823–0.904)	9.672 (6.808–13.741)	0.804
	Control type	HC	0.601 (0.554–0.647)	0.915 (0.895–0.932)	15.1337 (11.413–20.067)	0.844
		NCD	0.672 (0.617–0.723)	0.809 (0.737–0.865)	9.188 (6.525–12.934)	0.774
	Sample size	>=100	0.598 (0.552–0.643)	0.903 (0.884–0.919)	13.760 (10.676–17.734)	0.849
		<100	0.662 (0.544–0.762)	0.819 (0.694–0.900)	7.445 (4.461–12.425)	0.759
	Sample source	Plasma	0.620 (0.570–0.667)	0.884 (0.859–0.905)	12.547 (9.745–16.155)	0.831
		Serum	0.563 (0.477–0.645)	0.920 (0.873–0.951)	13.108 (7.187–23.906)	0.817
	Assay method	MSP	0.594 (0.551–0.636)	0.901 (0.882–0.917)	13.031 (10.216–16.623)	0.838
		Other	0.781 (0.611–0.890)	0.685 (0.482–0.836)	8.531 (3.834–18.981)	0.800
	Methylation gene location	SEPT-9	0.679 (0.622–0.732)	0.904 (0.881–0.923)	20.551 (14.684–28.760)	0.883
		Other	0.577 (0.522–0.631)	0.885 (0.854–0.910)	10.297 (7.750–13.681)	0.802
SEPT9 methylation Assay	Region	Asia	0.679 (0.616–0.737)	0.922 (0.902–0.938)	26.096 (17.252–39.471)	0.904
		Other	0.684 (0.572–0.777)	0.874 (0.824–0.917)	14.379 (8.808–23.472)	0.867
	Control type	HC	0.676 (0.609–0.737)	0.927 (0.900–0.947)	25.800 (15.869–41.945)	0.887
		NCD	0.723 (0.650–0.783)	0.859 (0.803–0.900)	17.164 (11.237–26.218)	0.854
	Sample size	≥100	0.688 (0.629–0.741)	0.908 (0.884–0.927)	21.949 (15.393–31.298)	0.888
		<100	0.603 (0.403–0.773)	0.832 (0.743–0.894)	10.289 (3.742–28.293)	0.847
	Sample source	Plasma	0.693 (0.631–0.748)	0.906 (0.883–0.925)	22.488 (16.257–31.105)	0.893
		Serum	0.575 (0.467–0.676)	0.880(0.750–0.947)	9.253 (2.297–37.269)	0.735

Abbreviations: 95% CI, 95% confidence interval; AUC, the area under the curve; CRC, colorectal cancer; ctDNA, circulating tumor DNA; DOR, diagnostic odds ratio; HC, healthy control; MSP, methylation-specific polymerase chain reaction; NCD, non-CRC disease; RT-PCR, real-time quantitative polymerase chain reaction; SEN, sensitivity; SPE, specificity.

**Table 3:** Meta-regression of impacts of study features on diagnostic value of ctDNA for CRC.

Analysis	Covariates	Coefficient	SE	*p*-Value
Quantitative analysis	Region	0.229	1.107	0.836
	Control type	0.607	1.268	0.632
	Sample size	1.467	1.333	0.271
	Sample source	−0.544	1.057	0.606
	Assay method	0.038	1.163	0.974
Qualitative analysis	Region	0.422	0.255	0.067
	Control type	0.502	0.237	0.034
	Sample size	0.504	0.357	0.158
	Sample source	−0.192	0.313	0.541
	Assay method	−0.122	0.482	0.800
	Methylation gene location	0.661	0.255	0.010
SEPT9	Region	0.676	0.322	0.045
	Control type	0.733	0.348	0.035
	Sample size	0.171	0.637	0.788
	Sample source	0.989	0.638	0.079

Abbreviations: CRC, colorectal cancer; ctDNA, circulating tumor DNA; SE, standard error.

## Data Availability

Not applicable.

## Supplementary Materials

The following supporting information can be downloaded at: https://www.mdpi.com/article/10.3390/jcm12020408/s1, Table S1: Summary of most relevant features of the enrolled publications. Figure S1: Traffic-light plot of quality assessment by the revised QUADAS-2. QUADAS-2, Quality Assessment of Diagnostic Accuracy Studies-2.
