# Supplementary material for: Using Circulating Tumor DNA as a Novel Biomarker to Screen and Diagnose Colorectal Cancer: A Meta-Analysis
Source: J Clin Med. 2023 Jan 4;12(2):408. doi: 10.3390/jcm12020408 (PMC9860998; doi:10.3390/jcm12020408)
Supplement: Supplementary file 1 [file jcm-12-00408-s001.zip › jcm-2080448-supplementary.pdf]

Table S1. Summary of the most relevant features of the included publications

First author，year	Country	Study type	Control type	Number of CRC/NCD/HC	Sample source	Sample timing	Detection methods	Detection indicators	Cutoff value	TP	FP	FN	TN	SEN (%)	SPE (%)	PLR	NLR
Ahlquist,2012	USA	NA	AD/HC	30/22/48	Plasma	Pre surgery	MSP	Methylation (*SEPT9*)	NA	18	16	12	54	60.0	77.1	2.625	0.519
Alizadeh,2018	Iran	NA	HC	30/0/40	Serum	Pre treatment	MSP	Methylation (*MGMT*)	NA	27	0	3	40	90.0	100.0	Infinity	0.100
Amiot,2014	France	NA	AD/HC	90/34/123	Serum	Pre surgery	MSP	Methylation (*WIF1*)	NA	29	2	61	155	32.2	98.7	25.294	0.687
Ansar, 2020	Taiwan	NA	HC	14/-/14	Plasma	Pre surgery	MSP	Methylation (*SMAD3*)	NA	11	6	3	8	78.6	57.1	1.833	0.375
Ashoori,2018	Iran	NA	HC	59/-/37	Plasma	NA	MSP	Methylation (*BMP3*)	NA	44	11	15	26	74.6	70.3	2.508	0.362
Bartak,2017	Hungary	NA	AD/HC	47/37/37	Plasma	NA	MSP	Methylation (*SFRP1*)	NA	40	40	7	34	85.1	45.9	1.574	0.324
								Methylation (*SFRP2*)	NA	34	35	13	39	72.3	52,7	1.529	0.525
								Methylation (*SDC2*)	NA	42	31	5	43	89.4	58.1	2.133	0.183
								Methylation (*PRIMA1*)	NA	38	36	9	38	80.9	51.4	1.662	0.373
Bedin,2017	Italy	Retrospective	AD/HC	70/18/36	Plasma	Pre treatment	MSP	Methylation (*OSMR*)	NA	31	8	39	46	44.3	85.2	2.989	0.654
								Methylation (*SFRP1*)	NA	44	6	26	48	62.9	88.9	5.657	0.418

First author，year	Country	Study type	Control type	Number of CRC/NCD/HC	Sample source	Sample timing	Detection methods	Detection indicators	Cutoff value	TP	FP	FN	TN	SEN (%)	SPE (%)	PLR	NLR
Cassinotti, 2012	USA	Prospective	AD/HC	30/30/30	Plasma	Pre surgery	Microarray	Methylation (*RASSF1A*)	NA	28	37	2	23	93.3	38.3	1.514	0.174
								Methylation (*HIC1*)	NA	19	11	11	49	63.3	81.7	3.455	0.449
								Methylation (*CYCD2*)	NA	29	42	1	18	96.7	30.0	1.381	0.111
								Methylation (*PAX5*)	NA	26	38	4	22	86.7	36.7	1.368	0.364
								Methylation (*RB1*)	NA	27	33	3	27	90.0	45.0	1.636	0.222
								Methylation (*SRBC*)	NA	10	5	20	55	33.3	91.7	4.000	0.727
Chen,2017	Taiwan	Prospective	HC	51/-/9	Plasma	NA	MSP	Methylation (*SEPT9*)	NA	24	1	27	8	47.1	88.9	4.235	0.596
Chen,2019	China	NA	HC	111/-/114	Serum	NA	MSP	Methylation (*SEPT9*)	NA	81	5	30	109	73.0	95.6	16.638	0.283
								Methylation (*SDC2*)	NA	79	5	32	109	71.2	95.6	16.227	0.302
Cho, 2020	South Korea	NA	HC	117/-/60	Plasma	Pre surgery	MSP and ddMethyLight	Methylation (*TMEM90B*)	1 Methylated Droplet	61	11	56	49	52.1	81.7	2.844	0.586
								Methylation (*PCDHG*)	1 Methylated Droplet	57	11	60	49	48.7	81.7	2.657	0.628
								Methylation	1 Methylated	55	8	62	52	47.0	86.7	3.526	0.611

First author，year	Country	Study type	Control type	Number of CRC/NCD/HC	Sample source	Sample timing	Detection methods	Detection indicators	Cutoff value	TP	FP	FN	TN	SEN (%)	SPE (%)	PLR	NLR
								(*PPP1R16B*)	Droplet								
								Methylation (*ADGRB1*)	1 Methylated Droplet	55	17	62	43	47.0	71.7	1.659	0.739
								Methylation (*GLI3*)	1 Methylated Droplet	51	4	66	56	43.6	93.3	6.538	0.604
								Methylation (*ANKRD13B*)	1 Methylated Droplet	48	7	69	53	41.0	88.3	3.516	0.668
								Methylation (*THBD*)	1 Methylated Droplet	46	13	71	47	39.3	78.3	1.815	0.775
								Methylation (*c9orf50*)	1 Methylated Droplet	37	9	80	51	31.6	85.0	2.108	0.804
								Methylation (*FAM123A*)	1 Methylated Droplet	27	2	90	58	23.1	96.7	6.923	0.796
								Methylation (*SLIT3*)	1 Methylated Droplet	26	3	91	57	22.2	95.0	4.444	0.819
Church,2014	USA, Germany	Prospective	AD/HC	53/523/934	Plasma	Pre treatment	MSP	Methylation (*SEPT9*)	NA	27	126	26	1331	50.9	91.4	5.891	0.537
deVos,2009	USA	NA	HC	97/-/172	Plasma	NA	MSP	Methylation (*SEPT9*)	NA	73	23	24	149	75.3	86.6	5.628	0.286
Fu,2018	China	NA	NCD/HC	98/131/253	Plasma	Pre surgery	MSP	Methylation (*SEPT9*)	NA	60	14	38	370	61.2	96.4	16.793	0.402
Grutzmann, 2008	USA	Prospective	HC	252/-/102	Plasma	NA	MSP	Methylation (*SEPT9*)	NA	120	7	132	95	47.6	93.1	6.939	0.562
He,2018	China	Prospective	NCD/HC	300/292/568	Plasma	Pre	MSP	Methylation	NA	221	70	79	790	73.7	91.9	9.050	0.287

First author，year	Country	Study type	Control type	Number of CRC/NCD/HC	Sample source	Sample timing	Detection methods	Detection indicators	Cutoff value	TP	FP	FN	TN	SEN (%)	SPE (%)	PLR	NLR
						treatment		(*SEPT9*)									
He,2010	China	NA	HC	182/-/170	Plasma	NA	MSP	Methylation (*ALX4*)	NA	87	11	95	159	47.8	93.5	7.388	0.558
								Methylation (*SEPT9*)	NA	136	6	46	164	74.7	96.5	21.172	0.262
								Methylation (*TMEFF2*)	NA	129	8	53	162	70.9	95.3	15.062	0.306
Herbst,2011	Germany	NA	HC	45/-/16	Serum	NA	MSP	Methylation (*NEUROG1*)	NA	25	3	20	13	55.6	81.3	2.963	0.547
								Methylation (*ALX4*)	NA	21	7	24	9	46.7	56.3	1.067	0.948
								Methylation (*SEPT9*)	NA	21	3	24	13	46.7	81.3	2.489	0.656
								Methylation (*Vimentin A*)	NA	14	6	29	10	32.6	62.5	0.868	1.079
Jensen,2019	Denmark	NA	HC	113/-/87	Plasma	Pre surgery	MSP	Methylation (*C9orf50*)	NA	86	8	27	79	76.1	90.8	8.277	0.263
								Methylation (*KCNQ5*)	NA	94	4	19	83	83.2	95.4	18.093	0.176
								Methylation (*CLIP4*)	NA	87	1	26	86	77.0	98.9	66.982	0.233
Jin,2015	China	NA	AD and HP /HC	135/250/91	Plasma	Pre treatment	MSP	Methylation (*SEPT9*)	NA	101	43	34	298	74.8	87.4	5.933	0.228
Johnson,2014	USA	Prospective	AD and HP	101/106/94	Plasma	Pre surgery	MSP	Methylation	NA	74	37	27	163	73.3	81.5	3.960	0.328

First author，year	Country	Study type	Control type	Number of CRC/NCD/HC	Sample source	Sample timing	Detection methods	Detection indicators	Cutoff value	TP	FP	FN	TN	SEN (%)	SPE (%)	PLR	NLR
			/HC					(*SEPT9*)									
Kang,2014	China	NA	HC	80/-/52	Plasma	NA	MSP	Methylation (*SEPT9*)	NA	60	1	20	51	75.0	98.1	39.000	0.255
Karam,2019	Egypt	NA	HC	65/-/70	Serum	NA	MSP	Methylation (*P16*)	NA	36	1	29	69	55.4	98.6	38.769	0.453
Kim,2018	South Korea	Prospective	AD	111/5/-	Plasma	Pre treatment	MSP	Methylation (*SEPT9*)	NA	44	0	67	5	0.396	1.000	Infinity	0.604
Lee,2013	South Korea	NA	HC	101/-/96	Plasma	Pre treatment	MSP	Methylation (*SEPT9*)	NA	37	9	64	87	36.6	90.6	3.908	0.699
Li,2019	China	NA	HC	62/-/55	Serum	NA	MSP	Methylation (*SFRP2*)	NA	43	7	19	48	69.4	87.3	5.449	0.351
Lin, 2021	China	NA	HC	402/-/402	Plasma	Pre treatment	ddPCR	Methylation (*MYO1-G*)	10% methylation ratio	339	22	63	380	84.3	94.5	15.409	0.166
Liu,2013	Singapore	Prospective	HC	37/-/20	Serum	Pre surgery	MSP	Methylation (*SEPT9*)	NA	20	2	17	18	54.1	90.0	5.405	0.511
Liu,2020	China	NA	AD/HC	90/13/81	Plasma	NA	MSP	Methylation (*SEPT9*)	NA	77	12	13	82	85.6	87.2	6.702	0.166
Liu, Z. 2021	China	NA	NCD and HC	120/60/63	Serum	Pre treatment	MSP	Methylation (*CLIP4*)	NA	92	19	28	104	76.7	84.6	4.963	0.276
Lofton,2008	USA	NA	HC	133/-/179	Plasma	NA	MSP	Methylation (*TMEFF2*)	NA	86	55	47	124	64.7	69.3	2.104	0.510
								Methylation (*NGFR*)	NA	68	29	65	150	51.1	83.8	3.156	0.583

First author，year	Country	Study type	Control type	Number of CRC/NCD/HC	Sample source	Sample timing	Detection methods	Detection indicators	Cutoff value	TP	FP	FN	TN	SEN (%)	SPE (%)	PLR	NLR
								Methylation (*SEPT9*)	NA	92	25	41	154	69.2	86.0	4.953	0.358
Loomans-Kropp, 2022	USA	NA	HC	27/-/87	Plasma	Pre surgery	MSP	Methylation (*SEPT9*)	Ct<45	24	8	3	79	88.9	90.8	9.667	0.122
Lu, 2022	China	Retrospective	NCD	179/147/-	Plasma	NA	MSP	Methylation (*SEPT9*)	Ct<41	137	17	42	130	76.5	88.4	6.618	0.265
Luo,2020	China	Prospective	NCD/HC	29/452/1012	Plasma	NA	MSP	Methylation (*cg10673833*)	NA	26	194	3	1270	89.7	86.7	6.766	0.119
Nagai,2017	Japan	NA	HC	114/-/53	Plasma	Pre surgery	MSP	Methylation (*LINE-1*)	0.36※	75	5	39	48	65.8	90.6	6.974	0.378
Oh,2013	South Korea	NA	HC	131/-/125	Serum	NA	MSP	Methylation (*SDC2*)	NA	114	6	17	119	87.0	95.2	18.130	0.136
Otero,2020	Spain	Prospective	NCD/HC	57/159/171	Serum	NA	MSP	Methylation (*NEUROG1*)	1.3518%※※	19	31	38	299	33.3	90.6	3.548	0.736
Pasha,2019	Egypt	NA	AD/HC	85/40/40	Serum	Pre treatment	MSP	Methylation (*RUNX3*)	NA	51	7	34	73	60.0	91.3	6.857	0.438
								Methylation (*SFRP1*)	NA	66	12	19	68	77.6	85.0	5.176	0.263
Pedersen, 2015	Australia	Retrospective	HC	74/-/144	Plasma	NA	MSP	Methylation (*BCAT1*)	NA	48	5	26	139	64.9	96.5	18.681	0.364
								Methylation (*IKZF1*)	NA	50	7	24	137	67.6	95.1	13.900	0.341
Pedersen, 2015	Australia and Netherlands	Prospective	NCD/HC	129/1522/450	Plasma	Pre surgery	MSP	Methylation (*BCAT1*)	NA	74	107	55	1865	57.4	94.6	10.572	0.451

First author，year	Country	Study type	Control type	Number of CRC/NCD/HC	Sample source	Sample timing	Detection methods	Detection indicators	Cutoff value	TP	FP	FN	TN	SEN (%)	SPE (%)	PLR	NLR
								Methylation (*IKZF1*)	NA	62	27	67	1945	48.1	98.6	35.103	0.527
Potter,2014	USA, Germany	Prospective	AD and HP /HC	44/1056/444	Plasma	NA	MSP	Methylation (*SEPT9*)	NA	30	318	14	1182	68.2	78.8	3.216	0.404
Rasmussen, 2017	Denmark	Retrospective	HC	193/-/102	Plasma	Pre treatment	MSP	Methylation (*ALX4*)	NA	55	1	138	101	28.5	99.0	29.067	0.722
								Methylation (*APC*)	NA	81	33	112	69	42.0	67.6	1.297	0.858
								Methylation (*NEUROG1*)	NA	40	20	153	82	20.7	80.4	1.057	0.986
								Methylation (*RASSF1A*)	NA	22	16	171	86	11.4	84.3	0.727	1.051
								Methylation (*SDC2*)	NA	47	6	146	96	24.4	94.1	4.140	0.804
								Methylation (*SEPT9*)	NA	47	5	146	97	24.4	95.1	4.968	0.795
								Methylation (*SFRP1*)	NA	42	7	151	95	21.8	93.1	3.171	0.840
								Methylation (*SFRP2*)	NA	39	18	154	84	20.2	82.4	1.145	0.969
								Methylation (*WIF1*)	NA	19	4	174	98	9.8	96.1	2.510	0.938
Rezvani,2017	Iran	NA	HC	37/-/37	Plasma	Pre treatment	MSP	Methylation (*SPG20*)	NA	30	1	7	36	81.1	97.3	30.000	0.194

First author，year	Country	Study type	Control type	Number of CRC/NCD/HC	Sample source	Sample timing	Detection methods	Detection indicators	Cutoff value	TP	FP	FN	TN	SEN (%)	SPE (%)	PLR	NLR
Rokni,2018	Iran	Retrospective	HC	45/-/50	Plasma	Pre treatment	MSP	Methylation (*BMP3*)	NA	18	3	27	47	40.0	94.0	6.667	0.638
Sakamoto, 2010	Japan	NA	AD/HC	51/10/10	Serum	Pre surgery	IP-MSP	Methylation (*p16*)	NA	22	0	29	20	43.1	100	Infinity	0.569
Salehi,2015	Iran	NA	HC	25/-/25	Serum	NA	MSP	Methylation (*ALX4*)	NA	17	3	8	22	68.0	88.0	5.667	0.364
Song,2018	China	NA	AD and HP /HC	371/272/610	Plasma	NA	MSP	Methylation (*SEPT9*)	NA	281	74	90	808	75.7	91.6	9.028	0.265
Suehiro,2018	Japan	NA	AD/HC	18/95/25	Serum	NA	CORD PCR	Methylation (*TWIST1*)	2.8 copies	8	32	10	88	44.4	73.3	1.667	0.758
Takane,2014	Japan	Prospective	NCD	120/96/-	Plasma	NA	MSP	Methylation (*PPP1R3C*)	NA	97	18	23	78	80.8	81.3	4.311	0.236
								Methylation (*EFHD1*)	NA	74	21	45	75	62.2	78.1	2.843	0.484
Tang,2011	China	NA	AD and HP /HC	169/109/30	Plasma	Pre treatment	MSP	Methylation (*SFRP2*)	NA	113	5	56	134	66.9	96.4	18.588	0.344
Tanzer,2010	Germany	NA	AD and HP /HC	33/127/34	Plasma	Pre treatment	MSP	Methylation (*SEPT9*)	NA	24	53	9	118	72.7	69.0	2.346	0.395
Tanzer,2010	Germany	NA	AD and HP /HC	5/49/22	Plasma	Pre treatment	MSP	Methylation (*ALX4*)	NA	2	26	3	45	40.0	63.4	1.092	0.947
Tóth,2012	Hungary	NA	HC	92/-/92	Plasma	Pre treatment	MSP	Methylation (*SEPT9*)	NA	73	1	19	91	79.3	98.9	73.000	0.209
Tóth,2014	Hungary	NA	AD/HC	34/26/24	Plasma	Pre treatment	MSP	Methylation (*SEPT9*)	PMR >0.01%	30	10	4	40	88.2	80.0	4.412	0.147

First author，year	Country	Study type	Control type	Number of CRC/NCD/HC	Sample source	Sample timing	Detection methods	Detection indicators	Cutoff value	TP	FP	FN	TN	SEN (%)	SPE (%)	PLR	NLR
Wang,2008	China	NA	NCD/HC	45/30/30	Serum	Pre treatment	MSP	Methylation (*RASSF1A*)	NA	13	2	32	58	28.9	96.7	8.667	0.736
Warren,2011	USA Russia	Retrospective	HC	50/-/94	Plasma	NA	MSP	Methylation (*SEPT9*)	6.25pg/mL	45	11	5	83	90.0	88.3	7.691	0.113
Wu,2016	China	Prospective	NCD/HC	291/438/295	Plasma	NA	MSP	Methylation (*SEPT9*)	Ct=41	223	43	68	690	76.6	94.1	13.063	0.248
Wu,2011	China	NA	NCD	85/45/-	Plasma	Pre treatment	MSP	Methylation (*DLC1*)	NA	36	4	49	41	42.4	91.1	4.765	0.633
Xie,2018	China	NA	NCD/HC	123/95/19	Plasma	Pre treatment	MSP	Methylation (*SEPT9*)	NA	76	9	47	105	61.8	92.1	7.827	0.415
Xu, F, 2021	China	NA	NCD	104/130/-	Plasma	Pre treatment	MSP	Methylation (*SEPT9*)	Ct=41.9	87	7	17	123	83.7	94.6	15.536	0.085
								Methylation (*SDC2*)	Ct=44.5	80	12	24	118	76.9	90.8	8.333	0.157
								Methylation (*BCAT1*)	Ct=45.0	87	8	17	122	83.7	93.8	13.594	0.096
Xue,2017	China	NA	HC	95/-/47	Plasma	Pre treatment	BSP	Methylation (*CBS* promoter)	NA	61	10	34	37	64.2	78.7	3.018	0.455
Yang,2019	China	Retrospective	HC	300/-/54	Serum	Pre treatment	MSP	Methylation (*SEPT9*)	NA	157	14	143	40	52.3	74.1	2.019	0.644
Young, 2021	Denmark, Australia, Netherlands and Russia	Prospective	NCD/HC	184/868/568	Plasma	Pre surgery	MSP	Methylation (*BCAT1*)	NA	87	86	97	1350	47.3	94.0	7.895	0.561

First author，year	Country	Study type	Control type	Number of CRC/NCD/HC	Sample source	Sample timing	Detection methods	Detection indicators	Cutoff value	TP	FP	FN	TN	SEN (%)	SPE (%)	PLR	NLR
								Methylation (*IKZF1*)	NA	109	76	75	1360	59.2	94.7	11.193	0.430
								Methylation (*IRF4*)	NA	92	46	92	1390	50.0	96.8	15.609	0.517
Yuan,2016	China	NA	AD/HC	187/25/109	Plasma	Pre treatment	MSP	Methylation (*SEPT9*)	NA	117	12	70	122	62.6	91.0	6.987	0.411
								Methylation (*OSMR*)	NA	140	20	47	114	74.9	85.1	5.016	0.295
Zhang,2015	China	NA	AD/HC	57/30/47	Plasma	Pre treatment	MSP	Methylation (*GATA5*)	NA	35	23	22	54	61.4	70.1	2.056	0.550
								Methylation (*SFRP2*)	NA	31	25	26	52	54.4	67.5	1.675	0.675
								Methylation (*ITGA4*)	NA	21	18	36	59	36.8	76.6	1.576	0.824
Zhao,2019	China	NA	HP/HC	117/78/166	Plasma	NA	MSP	Methylation (*SEPT9*)	NA	96	18	21	226	82.1	92.6	11.123	0.194
								Methylation (*SDC2*)	NA	81	16	36	228	69.2	93.4	10.558	0.329
Zhao,2020	China	NA	HC	122/-/91	Plasma	NA	MSP	Methylation (*SEPT9*)	NA	77	9	45	82	63.1	90.1	6.382	0.409
								Methylation (*SDC2*)	NA	69	4	53	87	56.6	95.6	12.867	0.454
Zheng,2011	China	NA	NCD/HC	65/20/20	Serum	Pre treatment	MSP	Methylation (*RUNX3*)	NA	27	2	38	38	41.5	95.0	8.308	0.615

First author, year	Country	Study type	Control type	Number of CRC/NCD/HC	Sample source	Sample timing	Detection methods	Detection indicators	Cutoff value	TP	FP	FN	TN	SEN (%)	SPE (%)	PLR	NLR
Zou,2002	China	NA	AD/HC	52/34/10	Serum	Pre surgery	MSP	Methylation (*p16*)	NA	14	0	38	44	26.9	100.0	Infinity	0.731
Agah,2017	Iran	NA	HC	74/-/36	Plasma	NA	RT-PCR	ctDNA	42.8ng/mL	60	9	14	27	81.1	75.0	3.243	0.252
Agostini,2011	Italy	NA	HC	67/-/35	Plasma	Pre surgery	RT-qPCR	Alu247	2.00ng/mL	63	0	4	35	94.0	100.0	Infinity	0.060
Allegretti, 2020	Italy	Prospective	HC	39/0/10	Plasma	Pre surgery	dPCR	ctDNA	NA	20	0	19	10	51.3	100	Infinity	0.487
Czeiger,2011	Israel	NA	HC	38/-/34	Serum	Pre surgery	Fluore-scent	ctDNA	841ng/mL	16	2	22	32	42.1	94.1	7.158	0.615
Danese,2010	Italy	NA	HC	118/-/26	Serum	Pre treatment	RT-PCR	ctDNA	37.0ng/mL	98	2	20	24	83.1	92.3	10.797	0.184
El-Gayar,2016	Egypt	NA	HC	50/-/20	Serum	Pre treatment	qPCR	ctDNA	3.3ng/μL	34	7	16	13	68.0	65.0	1.943	0.492
Flamini,2006	Italy	Retrospective	HC	75/-/75	Serum	Pre treatment	qPCR	ctDNA	12.5ng/mL	61	20	14	55	81.3	73.3	3.050	0.255
Hao,2014	China	Prospective	HC	104/-/110	Serum	NA	RT-qPCR	Alu115	694ng/mL	72	1	32	109	69.2	99.1	76.154	0.311
Junca,2020	France	Prospective	AD and HP /HC	20/70/40	Plasma	Pre treatment	ddPCR	ctDNA	12ng/mL	13	28	7	82	65.0	74.5	2.554	0.470
Lan,2017	Taiwan	NA	HC	329/-/95	Plasma	Pre surgery	qPCR	ctDNA	2700 copies/mL	272	4	57	91	82.7	95.8	19.635	0.181
Nagai,2017	Japan	NA	HC	114/-/53	Plasma	Pre surgery	qPCR	ctDNA	10.7ng/mL	60	13	54	40	52.6	75.5	2.146	0.628
Qi,2013	China	NA	HP/HC	31/30/92	Serum	Pre surgery	bDNA	Alu	634.9ng/mL	20	2	11	91	64.5	97.8	30.000	0.363

Abbreviations: AD, adenoma; CRC, Colorectal cancer; ctDNA, circulating tumor DNA; ddPCR, droplet digital polymerase chain reaction; FN, false negative; FP, false positive; HC, healthy control; HP, hyperplastic polyp; MSP, methylation-specific polymerase chain reaction; NA, not applicable; NCD, non-CRC

disease; NLR, negative likelihood ratio; PLR, positive likelihood ratio; qPCR, quantitative polymerase chain reaction; RT-PCR/RT-qPCR, real-time quantitative polymerase chain reaction; SEN, sensitivity; SPE, specificity; TN, true negative; TP, true positive.

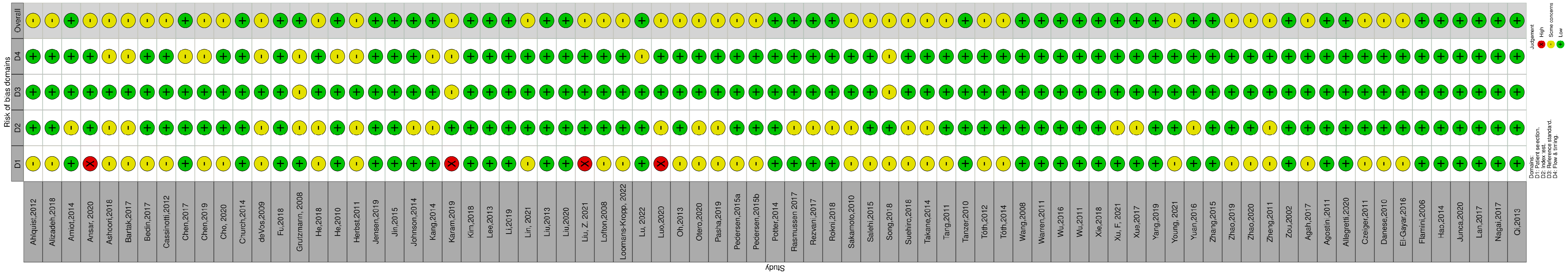

Figure S1. Quality assessment of the included studies by the revised QUADAS-2. QUADAS-2, Quality Assessment of Diagnostic Accuracy Studies-2.
